# A Comparison of Patient Satisfaction with Emergency Department Opt-In and Opt-Out Rapid HIV Screening

**DOI:** 10.1155/2012/904916

**Published:** 2012-02-16

**Authors:** Douglas A. E. White, Alicia N. Scribner, Maria E. Martin, Stacy Tsai

**Affiliations:** Department of Emergency Medicine, Alameda County Medical Center, Highland Hospital, Oakland, CA 94602, USA

## Abstract

*Study objective*. To compare patient satisfaction with emergency department (ED) opt-in and opt-out HIV screening. *Methods*. We conducted a survey in an urban ED that provided rapid HIV screening using opt-in (February 1, 2007–July 31, 2007) and opt-out (August 1, 2007–January 31, 2008) approaches. We surveyed a convenience sample of patients that completed screening in each phase. The primary outcome was patient satisfaction with HIV screening. *Results*. There were 207 and 188 completed surveys during the opt-in and opt-out phases, respectively. The majority of patients were satisfied with both opt-in screening (95%, 95% confidence interval [CI] = 92–98) and opt-out screening (94%, 95% CI = 89–97). Satisfaction ratings were similar between opt-in and opt-out phases even after adjusting for age, gender, race/ethnicity, and test result (adjusted odds ratio 1.3, 95% CI = 0.5–3.1). *Conclusions*. Emergency department patient satisfaction with opt-in and opt-out HIV screening is similarly high.

## 1. Introduction

### 1.1. Background

In 2006 the Centers for Disease Control and Prevention (CDC) published revised recommendations for HIV testing in health-care settings, which included emergency departments (EDs) [1]. Prior to these recommendations, the standard approach included opt-in HIV screening (in which patients are offered an HIV test and assent is required), separate written consent, and pre- and posttest counseling. The revised recommendations include using an opt-out approach to screening and removing requirements for test counseling and separate written informed consent as strategies to reduce barriers to testing and to make testing a routine part of care. With opt-out HIV screening, patients are notified that HIV testing will be performed unless they decline and consent for testing is integrated into the general ED consent process. Pretest counseling and risk assessment are not recommended, and written informational materials can replace posttest counseling and risk reduction strategies for patients that test negative. Access to clinical care and support services continue to be essential for patients with positive HIV test results [1].

The CDC recommends an opt-out approach for several reasons. By integrating opt-out screening into general consent, the screening process is streamlined and routinized. Patients may perceive the process to be less stigmatizing because they do not feel “singled out” for testing [1, 2]. It is hoped that adopting opt-out screening methodologies will increase screening rates. This is supported by the finding that, in some clinical settings, screening rates are higher with opt-out than with opt-in screening [3–6].

Despite the backing of the CDC and these initial clinical successes, some experts cite concerns with an opt-out approach to HIV screening [2, 7–12]. These concerns include inadequate patient preparation for testing, insufficient pretest information and counseling, coercion to test, loss of patient autonomy, and inappropriate testing of patients that lack the capacity to consent.

Patient satisfaction and attitudes toward opt-out HIV screening compared with opt-in HIV screening have not been studied. Understanding the experience of patients with opt-out screening is important for the successful integration of the revised CDC guidelines. The goal of this study was to compare patient satisfaction with ED HIV screening using an opt-in versus an opt-out approach.

## 2. Methods

### 2.1. Study Setting and Population

The study was conducted at an urban teaching hospital and regional trauma center in Oakland, California. In 2006, the annual ED census was approximately 75,000 visits; 47% of patients presenting to the ED were black, 32% were Hispanic, 21% were white, 44% were female, 98% were adults ≥15 years of age, and 80% did not have health insurance.

### 2.2. Study Design

We conducted a survey study to compare ED patient satisfaction and patient attitudes toward opt-in and opt-out rapid HIV screening. This survey study was administered concurrently with a prospective observational study comparing the outcomes of two successive 6-month screening periods. During the first 6 months, nontargeted opt-in HIV screening was performed and during the second 6 months, nontargeted opt-out screening was performed [13]. The medical center's institutional review board approved this study.

### 2.3. HIV Screening Protocol

A detailed description of the HIV screening protocol has previously been published [14]. In brief, all ED patients first completed a rapid triage nurse assessment in the waiting room area. During both screening phases, medically stable patients were provided a pretest HIV information brochure (that included information about HIV transmission, rapid HIV testing, and risk reduction) and were then referred to registration. After completing registration, patients were directed to the triage area for a standard triage nurse evaluation prior to ED placement.

Two full-time medical assistants were hired as HIV testers and worked during the entire study period. HIV testers were located in a testing station within the triage area on weekdays between 7 am and 10 pm. Only one HIV tester was on duty at any given time. During hours when the HIV tester was not on site, screening was not performed. During both study phases, HIV testing was performed at the testing station using the OraQuick ADVANCE Rapid HIV 1/2 Antibody Test (OraSure Technologies, Inc. Bethlehem, PA, USA) on oral fluid specimens. HIV test results were documented in special fields in the electronic medical record (Wellsoft Corporation, Somerset, NJ, USA) accessible by clinical staff. Results were interpreted as reactive, negative, or indeterminate.

Patients were eligible for HIV screening if they were ≥15 years of age, medically stable, and able to consent for HIV testing (opt-in phase) or able to complete the general consent for care (opt-out phase).

### 2.4. Nontargeted Opt-In Screening Phase (February 1, 2007–July 31, 2007)

During this phase, all medically stable patients were referred by the triage nurse to the HIV tester. HIV testers determined eligibility and offered HIV screening using the following opt-in script: “Would you like to have a rapid HIV test today?” Patients opting-in completed a streamlined HIV consent form followed by immediate testing. No additional counseling was performed, and patients were directed to the waiting room after collection of an oral fluid sample.

### 2.5. Nontargeted Opt-Out Screening Phase (August 1, 2007–January 31, 2008)

During this phase, consent for HIV testing was integrated into the general consent form for medical care in accordance with CDC guidelines and California state law and completed during registration. The general ED consent form was modified to include both a statement that HIV testing may be performed during the ED visit unless the patient declined and an opt-out signature box next to the statement, “I do not want to be HIV tested.” Registration staff determined eligibility and offered HIV screening using the following opt-out script: “HIV testing may be performed during your emergency room visit, if you do not want to be HIV tested sign here.” Patients declined testing by signing in the opt-out signature box. Registration staff electronically flagged the charts of patients not opting-out and then referred these patients to the triage area where rapid testing was performed by the HIV testers.

### 2.6. HIV Result Disclosure

In both study phases, HIV test results were documented and provided to patients using the same procedures and printed materials. After tests were completed, the HIV testers placed an electronic order instructing the nurse to disclose negative results once the patient was placed in the ED. Nursing staff verbally disclosed the negative results and provided patients with a postresult informational handout that documented the result, explained risk reduction strategies and indications for repeat testing, and provided a list of testing resources. A copy of the postresult handout was also added to the patient's electronic discharge instructions.

Patients who had reactive HIV test results were placed immediately in a private room in the ED. HIV testers and ED physicians provided patients with a verbal explanation of the reactive test result, offered emotional support, and arranged direct linkage to follow-up care. A postresult informational handout was also provided that explained the preliminary nature of the test result, the importance of confirmatory testing and follow-up care, strategies to prevent transmission, and referral for mental health and other support services. Blood was drawn and sent for CD4 cell count, viral load, and Western blot testing. Drop-in follow-up appointments were available on select dates and times at the medical center's HIV clinic.

### 2.7. Study Protocol

The survey instrument was developed by one of the authors in collaboration with experts from the CDC and an emergency medicine physician with experience in ED-based rapid HIV testing (Appendix). The survey instrument was pilot tested on a convenience sample of patients (*n* = 10), and refinements were made based on feedback. The survey elicited demographic information, including age, gender, race/ethnicity, education level, type of health insurance, current relationship status, risk factors for HIV, previous HIV testing, satisfaction with the screening program, and attitudes toward the screening program. The target population for survey administration was all screened patients with reactive results and a convenience sample of screened patients with negative results.

The survey was administered to English and Spanish speaking patients with negative HIV screening tests during the last three months of the opt-in and opt-out screening phases (May 1, 2007–July 31, 2007 and November 1, 2007–January 31, 2008), respectively. The two survey administrators were college research volunteers who were trained in survey administration and who were not blinded to the study purpose. Working in three-hour shifts, survey administrators reviewed the ED electronic record and identified all patients who had completed HIV screening, received their test results, and had not been discharged. Patients were then approached, and eligible patients were provided an explanation of why the study was being performed and written informed consent was obtained. Patients were ineligible for survey administration according to prespecified criteria: unavailable, too ill, language spoken other than English/Spanish, or altered mental status/confusion. Determination of eligibility was at the discretion of the survey administrators. The survey was verbally administered face-to-face at the patient's bedside. In order to evaluate the satisfaction and attitudes of patients with reactive HIV screening tests, these patients were asked to participate in the study as part of the result disclosure process. Surveys for this subset of patients were conducted by a single research coordinator.

### 2.8. Outcome Measures

The primary outcome measure was overall satisfaction with opt-in and opt-out screening. The global index of satisfaction was determined by asking the patient, “Overall, how would you rate the rapid HIV testing program in the emergency department?” Responses were graded on a 5-point Likert scale (poor, fair, good, very good, and excellent). Respondents were classified as satisfied with the HIV testing program if they rated the program good, very good, or excellent and dissatisfied if they rated the program poor or fair. Secondary outcome measures were the attitudes of patients toward each screening program. The attitudes of patients toward the screening program were evaluated by assessing their level of agreement (agree, disagree) with 13 potential indicators of satisfaction over 6 domains. The domains were designed to evaluate (1) patient satisfaction, (2) coercion to test, (3) impact of testing on care received, (4) confidentiality of screening, (5) information provided, and (6) patient beliefs regarding the role of HIV screening. 

### 2.9. Data Analysis

The research volunteers entered the survey responses into a spreadsheet (Microsoft Excel 2003, Microsoft Corporation, Redmond, WA, USA). The first 25 surveys by each research volunteer were reviewed to ensure accurate entry of responses, and inconsistent data were identified and reconciled. The study population and results of the satisfaction survey are reported in descriptive statistics. Categorical data are reported as percentages with 95% confidence intervals (CIs). Frequencies of survey responses and demographics were evaluated by screening program (opt-in versus opt-out) using Chi-square analysis and Fisher's exact tests. We specified a logistic regression model to predict responses to questions about patient satisfaction and patient attitudes toward the opt-in and opt-out screening programs, based on consent arm allocation and adjusting for the variables age, gender, race/ethnicity, and HIV test result, which, because of each covariate's theoretic importance in the model, were included regardless of significance in bivariate testing. A priori sample size calculation was performed and based on an opt-in satisfaction rate of 95% (pilot data), 159 patients per group were needed to determine an absolute difference in satisfaction of 10% between opt-in and opt-out screening, assuming a *P* value < 0.05 and power 80%. All statistical analyses were performed using SPSS 16.0 (SPSS Inc., Chicago, IL, USA).

## 3. Results

**Figure 1:**
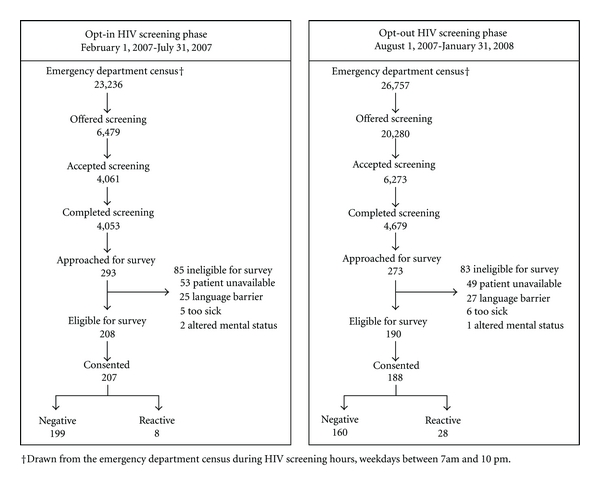
Study flow diagram.

The study flow is outlined in Figure 1. For the opt-in versus the opt-out screening phases, results were as follows: there were 23,236 potentially eligible patients versus 26,757; screening offer rate was 27.9% (6,479/23,236) versus 75.8% (20,280/26,757) (*P* < 0.001); screening acceptance rate was 62.7% (4,061/6,479) versus 30.9% (6,273/20,280) (*P* < 0.001); testing completion rate was 99.8% (4,053/4,061) versus 74.6% (4,679/6,273) (*P* < 0.001); overall screening rate was 17.4% (4,053/23,236) versus 17.5% (4,679/26,757) (*P* = 0.90).

During the opt-in phase, research volunteers approached 293 of the 4,053 patients that completed HIV screening to participate in the survey. Of these, 208 were eligible and 207 consented and completed the survey. During the opt-out phase, research volunteers approached 273 of the 4,679 patients that completed HIV screening to participate in the survey. Of these, 190 were eligible and 188 consented and completed the survey. Reasons patients were ineligible for survey administration were similar between both phases and included: patient unavailable (61%), language barrier (31%), too ill (7%), and altered mental status/confusion (2%). Of the 207 patients who completed surveys during the opt-in phase, 199 (96%) tested negative and 8 (4%) had reactive test results. Of the 188 patients who completed surveys during the opt-out phase, 160 (85%) tested negative and 28 (15%) had reactive test results. 

Characteristics of patients accepting screening and characteristics of patients surveyed in both opt-in and opt-out HIV screening phases are shown in Table 1. Patients accepting opt-in screening were similar to patients accepting opt-out screening with respect to age and gender; however, there were differences in race/ethnicity. There were no differences with respect to age, race/ethnicity, education level, HIV risk, or previous HIV testing between patients surveyed during the opt-in and the opt-out phases; however opt-out surveyed patients were more likely to be male, uninsured, and single. 

Patient satisfaction ratings were available for 393 of the 395 surveyed patients and are shown in Table 2. One patient testing negative in each phase chose the option “prefer not to answer/I do not know” when asked to rate their satisfaction. The majority of patients in the opt-in phase (95.1%, 95% CI = 92.0–98.0) and the opt-out phase (93.6%, 95% CI = 89.3–96.7) were satisfied with the HIV screening program. A higher proportion of patients who experienced opt-in screening rated the experience as excellent rather than very good, as compared to the opt-out ratings which were more frequently very good rather than excellent. Overall satisfaction ratings remained similar between opt-in and opt-out phases even after adjusting for age, gender, race/ethnicity, and HIV test result (adjusted odds ratio 1.3, 95% CI = 0.5–3.1, data not shown). 

Responses to questions assessing patient attitudes toward HIV screening are shown in Table 3. Among the domains, most attitudes towards opt-in and opt-out screening were similar. These similarities persisted after adjusting for age, gender, race/ethnicity, and HIV test result. Although a significantly higher proportion of patients during the opt-in phase would have liked additional discussion about HIV risk and HIV prevention, most patients in both phases felt that the information they were given about testing was adequate. The majority of patients in both phases reported being satisfied with the HIV testing received and would recommend the ED as a place to get HIV tested. Patients completing opt-out HIV screening did not report feeling any more pressured into testing and still felt like they had a choice about getting tested when compared with patients completing opt-in screening. Most patients also agreed that HIV testing did not interfere with the overall care received and perceived the testing process to be confidential. Over 90% of patients in both phases felt that HIV testing should be a regular part of health care and that routine testing should be performed in the ED.

## 4. Discussion 

The CDC has called for “explicit and measurable indicators to measure the progress on the process and outcomes” of the revised recommendations [15]. Since release of the 2006 recommendations, three ED studies have reported on specific outcomes associated with opt-out screening, including the number of persons tested, the number of patients receiving HIV care as a result of screening, baseline CD4 count, and number of false-positive screening test results [14, 16, 17]. This is the first study to focus on patient satisfaction and patient attitudes about HIV screening, and to compare these outcomes between opt-in and opt-out screening programs. Patient satisfaction with ED-based HIV screening may be an important determinant of a program's success. Not only may patient satisfaction influence acceptance rates but it may also affect the willingness of administrators and legislators to support screening programs. 

We demonstrate that patient satisfaction is similar with opt-in and opt-out HIV screening protocols in an urban ED. Over 95% of patients surveyed reported being satisfied with HIV testing and would recommend the ED as a testing site. Brown et al. also assessed patient perceptions with routine opt-out HIV screening and similarly showed that over 90% of ED patients surveyed would “recommend to a friend to get an HIV test in the ER” and that over 3/4 of patients felt that “the ER is a good place to perform screening” [18]. 

Patients in both screening phases rated their overall experience with HIV screening favorably. However, individuals surveyed during the opt-in phase of the study were more likely to report their overall experience as excellent. The reason for this difference between the two phases is unknown, but it may be explained by the extra time the HIV testers spent with the patients reviewing the consent form during the opt-in testing phase or the manner in which patients were consented for testing. 

We also demonstrate that patient attitudes over a wide variety of domains are similar between opt-in and opt-out HIV screening protocols. Importantly, patients did not report feeling coerced to test and maintained their autonomy in deciding whether to be tested, even with the opt-out methodology. Preserving patient perception of autonomy with opt-out HIV screening is important because this has been cited as a major concern with the revised CDC guidelines [7]. 

Additionally, this study provides more detailed data about patient satisfaction with streamlined ED screening than any prior study. The streamlined format eliminated pretest counseling (providing instead a pretest information brochure) and simplified negative posttest counseling. Despite the streamlined approach, patients were remarkably satisfied, and most patients felt the information provided to them was adequate. This is consistent with the results of another survey study that assessed ED patient acceptance and understanding of opt-in and opt-out HIV testing [19]. Of 529 respondents, approximately 2/3 felt that counseling was unnecessary both before testing and after receiving a negative HIV test. While the majority of patients in both study phases did not want additional counseling, patients surveyed during the opt-in phase were more likely to desire an in-depth discussion regarding HIV. The reason for this difference is not known; however, one possibility is that the more thorough consenting process with the HIV tester during the opt-in phase generated an additional desire for information in a small number of the patients surveyed. 

The generalizability of our results to other screening programs is not known. Our method of implementing opt-out HIV screening represents one of several methods. Because California state law requires documentation of patient refusal of HIV screening, we added an opt-out signature box to the general consent form—essentially creating an abbreviated written consent for patients to sign if they decline. Additionally, we chose to soften the opt-out language, instructing patients that HIV testing *may* be done unless they decline. This interpretation of opt-out screening may not be fully consistent with the intent of the CDC recommendations. Patient satisfaction and attitudes toward opt-out HIV screening may be somewhat different in institutions that implement different models of opt-out screening. 

This study has several limitations. Although the survey instrument was developed using standard methodology and pilot tested on a small convenience sample of subjects and refined with their feedback, the instrument has not been validated elsewhere. Additionally, content validity was not formally assessed, and participants may have misunderstood particular survey questions leading to biased results. 

Convenience sampling and the relatively small surveyed population (approximately 5% of screened patients) may have resulted in selection bias. Furthermore, not including patients who declined HIV screening may have influenced the findings, probably toward higher satisfaction ratings [18]. The research volunteers who administered the survey were not blinded to the study purpose, and the study was not self-administered. Although research assistants were trained to remain neutral when presenting the survey, the influence of the Hawthorne effect may have further biased respondents to favorable ratings. 

Because we evaluated only those patients who completed HIV screening, the results may lack generalizability. We did not assess how nontesters felt about the testing method, and we were therefore unable to determine whether the testing methodology influenced a patient's decision not to test. 

We did not evaluate patient comprehension of the opt-in and opt-out screening protocols, and we are unable to report whether patients misunderstood the opt-out consent process. Because we utilized point-of-care rapid HIV testing using dedicated HIV testers and oral fluid sampling, it is unlikely that patients were tested unknowingly—even if some patients may have initially misunderstood the consent process. This may not hold true for programs, however, that link acceptance of opt-out HIV screening at registration with testing on blood samples later obtained as a part of ED care. Determining patient comprehension of opt-out HIV screening, specifically when HIV consent is integrated into the general consent for care, should be evaluated in future studies to ensure that patients are not tested unknowingly. 

## 5. Conclusions

Our results suggest that, among the subset of ED patients surveyed, satisfaction with HIV screening is high, regardless of whether opt-in or opt-out screening is implemented. Furthermore, patients did not feel coerced into testing and patient autonomy was preserved even with opt-out methods and elimination of separate written consent. These results may encourage more widespread implementation of opt-out HIV screening in EDs.

## Figures and Tables

**Table 1:** Comparison of opt-in and opt-out patients.

Characteristic	Accepted opt-in screening (*n* = 4, 061), *n* (%)	Accepted opt-out screening (*n* = 6, 273), *n* (%)	*P* value^a^	Surveyed opt-in screening (*n* = 207), *n* (%)	Surveyed opt-out screening (*n* = 188), *n* (%)	*P* value^a^
Gender						
Male	2,103 (52)	3,319 (53)	0.264	102 (49)	119 (63)	0.005
Female	1,958 (48)	2,954 (47)		105 (51)	69 (37)	
Age (years), mean SD	38 ± 13	38 ± 13	>0.999^b^	37 ± 13	39 ± 13	0.128^b^
Race/ethnicity						
Black	1,625 (40)	2,862 (46)	<0.001	77 (37)	88 (47)	0.883
White	563 (14)	859 (14)		28 (14)	29 (15)	
Hispanic	1,262 (31)	1,826 (29)		77 (37)	53 (28)	
Other	611 (15)	726 (12)		25 (12)	18 (10)	
Education level						
Some high school	na	na		63 (30)	49 (26)	0.747
High school graduate	na	na		67 (32)	67 (36)	
Some college	na	na		52 (25)	53 (28)	
College degree	na	na		18 (9)	15 (8)	
Unknown	na	na		7 (3)	4 (2)	
Health insurance						
No insurance	na	na		141 (68)	145 (77)	0.076
Public insurance	na	na		45 (22)	35 (19)	
Private insurance	na	na		9 (4)	2 (1)	
Unknown	na	na		12 (6)	6 (3)	
Relationship status						
Single	na	na		97 (47)	119 (63)	0.009
Married/partnered	na	na		63 (30)	37 (20)	
Divorced/separated	na	na		46 (22)	32 (17)	
Unknown	na	na		1 (0.5)	0	
Reported risk past 12 months						
Heterosexual sex	na	na		142 (69)	126 (67)	0.532
MSM only	na	na		5 (2)	7 (4)	
IDU only	na	na		2 (1)	6 (3)	
MSM + IDU	na	na		1 (0.5)	1 (0.5)	
None	na	na		57 (28)	48 (26)	
Previous HIV test						
Yes	na	na		120 (58)	113 (60)	0.667
No	na	na		87 (42)	75 (40)	

^a^Pearson Chi-square.

^b^ *t*-test.

Abbreviations: na: not available; MSM: men sex with men; IDU: injection drug use.

**Table 2:** Patient satisfaction with HIV screening^a^, *n* = 393.

Screening	Excellent		Very good		Good		Fair		Poor	
	*n*	% (95% CI)	*n*	% (95% CI)	*n*	% (95% CI)	*n*	% (95% CI)	*n*	% (95% CI)
Opt-in										
All	130/206	63.1 (56.5–69.7)	30/206	14.6 (9.7–19.4)	36/206	17.5 (12.3–22.7)	9/206	4.37 (1.6–7.2)	1/206	0.5 (0–1.4)^b^
Negative	129/198	65.2 (58.5–71.8)	29/198	14.7 (9.7–19.6)	31/198	15.7 (10.6–20.7)	8/198	4.04 (1.3–6.8)	1/198	0.5 (0–1.5)^b^
Positive	1/8	12.5 (0–35.4)^a^	1/8	12.5 (0–35.4)^a^	5/8	62.6 (29.0–96.1)	1/8	12.50 (0–35.4)^b^	0/8	
Opt-out										
All	91/187	48.7 (41.5–55.8)	54/187	28.9 (22.4–35.4)	30/187	16.0 (10.8–21.3)	11/187	5.9 (2.5–9.3)	1/187	0.5 (0–1.6)^b^
Negative	83/159	52.2 (44.4–60.0)	45/159	28.3 (21.3–35.3)	21/159	13.2 (8.0–18.5)	10/159	6.3 (2.5–10.1)	0/159	
Positive	8/28	28.6 (11.8–45.3)	9/28	32.1 (14.8–49.4)	9/28	32.1 (14.8–49.4)	1/28	3.6 (0–10.4)	1/28	3.6 (0–10.4)^b^

^a^The global index of satisfaction was determined by asking the patient, “Overall, how would you rate the rapid HIV testing program in the emergency department?”

^b^1-sided, 97.5% CI.

Abbreviation: CI: confidence interval.

**Table 3:** Patient attitudes towards opt-in and opt-out HIV screening, *n* = 395.

Domain	Question	Screening phase	Agree		Disagree		OR	Adjusted OR^a^
			*n*	% (95% CI)	*n*	% (95% CI)	(95% CI)	(95% CI)
*Satisfaction*	I would recommend the ER to others as a good place to get tested for HIV	Opt-in	197/207	95.2 (92.3–98.1)	7/207	3.4 (0.9–5.8)	—	—
		Opt-out	183/188	97.3 (95.0–99.6)	0/188	0	—	—
	I was satisfied with the HIV testing that I received today	Opt-in	200/207	96.6 (94.2–99.1)	6/207	2.9 (0.6–5.2)	ref	ref
		Opt-out	180/188	95.7 (92.9–98.6)	4/188	2.1 (0.1–4.2)	1.4 (0.4–4.9)	2.0 (0.5–8.1)

*Coercion*	I felt pressured into getting an HIV test	Opt-in	18/207	8.7 (4.9–12.5)	181/207	87.4 (82.9–92.0)	ref	ref
		Opt-out	6/188	3.2 (0.7–5.7)	180/188	95.7 (92.9–98.6)	0.3 (0.1–0.9)	0.3 (0.1–0.8)
	I felt like I had a choice about getting an HIV test	Opt-in	194/207	93.7 (90.4–97.0)	6/207	2.9 (0.6–5.2)	ref	ref
		Opt-out	183/188	97.3 (95.0–99.6)	4/188	2.1 (0.1–4.2)	1.4 (0.4–5.1)	1.2 (0.3–4.4)

*Process of care*	I had to wait too long for my HIV test result	Opt-in	27/207	13.0 (8.5–17.6)	173/207	83.6 (78.5–88.6)	ref	ref
		Opt-out	17/188	9.0 (4.9–13.1)	168/188	89.4 (85.0–93.8)	0.6 (0.3–1.2)	0.7 (0.4–1.4)
	HIV testing interfered with the overall care I received in the ER	Opt-in	12/207	5.8 (2.6–9.0)	189/207	91.3 (87.5–95.1)	ref	ref
		Opt-out	13/188	6.9 (3.3–10.5)	173/188	92.0 (88.2–95.9)	1.2 (0.5–2.7)	0.9 (0.4–2.3)

*Confidentiality*	Overall, I felt that the HIV testing done today was private	Opt-in	191/207	92.3 (88.6–95.9)	6/207	2.9 (0.6–5.2)	ref	ref
		Opt-out	180/188	95.7 (92.9–98.6)	5/188	2.7 (0.4–5.0)	1.1 (0.3–3.8)	1.9 (0.5–7.0)
	I felt that my HIV test was told to me in a private way	Opt-in	196/207	94.7 (91.6–97.7)	5/207	2.4 (0.3–4.5)	ref	ref
		Opt-out	179/188	95.2 (92.2–98.3)	4/188	2.1 (0.1–4.2)	1.1 (0.3–4.3)	1.2 (0.3–4.8)

*Patient attitudes*	HIV testing should be a regular part of health care	Opt-in	200/207	96.6 (94.2–99.1)	3/207	1.4 (0–3.1)^b^	ref	ref
		Opt-out	182/188	96.8 (94.3–99.3)	3/188	1.6 (0–3.4)^b^	0.9 (0.2–4.6)	2.5 (0.4–16.5)
	Routine HIV testing should be performed in the ER	Opt-in	188/207	90.8 (86.9–94.8)	8/207	3.9 (1.2–6.5)	ref	ref
		Opt-out	182/188	96.8 (94.3–99.3)	3/188	1.6 (0–3.4)^b^	2.6 (0.7–9.9)	3.2 (0.7–13.8)

	I understand the meaning of my HIV test result	Opt-in	201/207	97.1 (94.8–99.4)	3/207	1.4 (0–3.1)^b^	ref	ref
		Opt-out	179/188	95.2 (92.2–98.3)	1/188	0.5 (0–1.6)^b^	2.7 (0.3–26.0)	2.6 (0.3–26.1)
*Information dissemination*	The information I was given about HIV testing was just about right	Opt-in	192/207	92.7 (89.2–96.3)	8/207	3.9 (1.2–6.5)	ref	ref
		Opt-out	172/188	91.4 (87.5–95.5)	4/188	2.1 (0.07–4.2)	1.8 (0.5–6.1)	3.0 (0.8–11.3)
	I would like to have a more in-depth discussion about my risks of getting HIV and ways to prevent it^c^	Opt-in	46/199	23.1 (17.3–29.0)	135/199	67.8 (61.4–74.3)	ref	ref
		Opt-out	15/160	9.4 (4.9–13.9)	134/160	83.8 (78.0–89.5)	3.0 (1.6–5.7)	3.2 (1.7–6.3)^d^

^a^Odds ratio adjusted for age, gender, race/ethnicity, and HIV test result.

^b^1-sided, 97.5% CI.

^c^Only asked of patients with negative test results, *n* = 199 Opt-in, *n* = 160 Opt-out.

^d^Odds ratio adjusted for age, gender, and race/ethnicity.

Frequencies were calculated as a fraction of the total data set and do not add up to 100% due to subjects choosing the response “prefer not to answer.”

Abbreviations: OR: odds ratio; ER: emergency room; CI: confidence interval; ref: reference category.
